# Description of olive morphological parameters by using open access software

**DOI:** 10.1186/s13007-017-0261-8

**Published:** 2017-12-11

**Authors:** Konstantinos N. Blazakis, Maria Kosma, George Kostelenos, Luciana Baldoni, Marina Bufacchi, Panagiotis Kalaitzis

**Affiliations:** 1 Department of Horticultural Genetics and Biotechnology, Mediterranean Agronomic Institute of Chania (MAICh), Alsyllio Agrokipiou, PO BOX 85, 73100 Chania-Crete, Greece; 2 Kostelenos Olive Nurseries, 18020 Poros-Trizinias, Greece; 3 Italian National Research Council, Institute of Biosciences and Bio-Resources (CNR-IBBR), Via Madonna Alta, 130-06128 Perugia, Italy; 4 Italian National Research Council, Institute for Agriculture and Forest Systems in the Mediterranean (CNR-ISAFOM), Via Madonna Alta, 130-06128 Perugia, Italy

**Keywords:** Morphological analysis, Image analysis, Olive, Fruit, Leaf, Endocarp, Morphological analysis of crop species

## Abstract

**Background:**

The morphological analysis of olive leaves, fruits and endocarps may represent an efficient tool for the characterization and discrimination of cultivars and the establishment of relationships among them. In recent years, much attention has been focused on the application of molecular markers, due to their high diagnostic efficiency and independence from environmental and phenological variables.

**Results:**

In this study, we present a semi-automatic methodology of detecting various morphological parameters. With the aid of computing and image analysis tools, we created semi-automatic algorithms applying intuitive mathematical descriptors that quantify many fruit, leaf and endocarp morphological features. In particular, we examined quantitative and qualitative characters such as size, shape, symmetry, contour roughness and presence of additional structures such as nipple, petiole, endocarp surface roughness, etc..

**Conclusion:**

We illustrate the performance and the applicability of our approach on Greek olive cultivars; on sets of images from fruits, leaves and endocarps. In addition, the proposed methodology was also applied for the description of other crop species morphologies such as tomato, grapevine and pear. This allows us to describe crop morphologies efficiently and robustly in a semi-automated way.

**Electronic supplementary material:**

The online version of this article (10.1186/s13007-017-0261-8) contains supplementary material, which is available to authorized users.

## Background

It is of great importance to evaluate and characterize the phenotypic diversity of crop species. Among them olive represents a significant species due to its rich patrimony of cultivars and wild plants [1, 2]. The great variability of the olive cultivars at the morphological level is easily detectable in fruit, leaf and endocarp shape and size. Therefore, their morphological analysis might represent an efficient tool for the characterization and discrimination of cultivars and the establishment of phenological relationships among them [3–6]. In recent years, much attention has been focused on the comparison of morphological characteristics of the main olive cultivars [7–9]. Despite this, the majority of the efforts have been concentrated on the molecular genotyping of cultivars in order to assess their genetic diversity [10–13].

This approach still continues to constitute the main way for the description and classification of the olive germplasm, despite numerous limitations, such as variability of the environmental condition, the age of the trees, the cultivation systems, and the phenological stage of the plants. Most of the studies based on morphological descriptors which have been adopted by the International Union for the Protection of New Varieties of Plants (UPOV) focus on the morphological characteristics of leaves, fruits and endocarps. Traditionally, these characteristics have been widely used for descriptive purposes to distinguish olive cultivars [3, 14–18]. Several descriptors which are related to leaf, fruit and endocarp traits have been shown to be mostly genetically determined with limited influence by the environment [19, 20]. Morphological characteristics are sometimes correlated or associated with disease susceptibility and can be used as markers in breeding for disease tolerance [21–23]. Despite the wide use of this approach for the identification of olive cultivars, there is a lack of a methodology to further assist the development of this field [6]. Thus, the morphological analysis of olive leaves, fruits and endocarps with computerised tools might be considered important for this field study in order to infer as much as we can from their numerical findings.

Moreover, the rapid development in imaging techniques has generated a large amount of discrete data from plants and plant organs, in general. However, the use and the inference from all these observations remains a challenge. The use of the imaging data and the computer-based techniques which new technologies can offer, it is of vital importance for the development of novel tools and algorithms for the description of organ morphology.

Currently old-fashioned manual techniques such as using screw gauge or calliper, gridded paper, etc. have been used for the morphological analysis of olives [5]. Moreover, additional methods were also used up to now which impose of some prerequisites (e.g. colour of the images background, etc.) were also used up to now. *ImageJ* [24] is one of the most commonly used, open access, scientific image analysis software. Parameters like the height, the width, the area or perimeter of the descriptive object, are easily extractable from the program.

To date, there is an increasing use of image analysis and computerised methodologies in plant science and agriculture, especially in the field of phenomics and there are plenty of tools for such purposes [25–27]. In addition, semi-automated computerised tools and software have been developed to perform morphological analysis of plant species. *Tomato Analyser* (TA) is one of them and is already being widely used to describe plant organ’s morphology [28–30]. Although this software has been designed to conduct phenotypic measurements of tomatoes, the developers suggested that can be applied for other crop species. LeafAnalyser is also a robust and effective automated tool developed only for leaves capable of measuring their shape [31]. The user can easily extract the margin of the leaf and perform all the associated calculations regarding its shape. Another computational tool was also developed which provides information on the leaf’s shape description [32]. *LAMINA* provides usual automated measurements related to leaf shape, size, asymmetry, but in addition, quantify leaf serration traits and herbivory damages. More recently, *SmartGrain* [33] was developed as a computerised platform for measuring the features of shape only for seeds. Furthermore, other software have been realised that can measure leaf outlines and extract individual leaf shape traits, including among others leaf height, leaf area and leaf width [34–38]. Although all the above applications can provide accurate measurements, there are some limitations in either complicated usage or to assess complex morphological traits, like tip shape or symmetry. Likewise, seed or fruit shape can be analysed with the software SHAPE [39] or GrainScan [40], but there are some restrictions in detecting important morphological traits, like for a example the presence of a fruit nipple or the seed roughness.

In addition to these software applications, purely geometrical and mathematical notions have been used for the morphological analysis of several plant species. Elliptic Fourier methodology has been applied to describe the morphology of the leaves, the fruits and the seeds [41–44]. This methodology provides basic measurements regarding the features of shape of plant organs by using advance mathematical skills. Moreover, a methodology was developed to classify defected olive fruits according to their external appearance [45]. This methodology, in addition to the identification of the wounded olive fruits, provided some basic morphological features only for the fruits such as height and perimeter. Finally, the needs of the plant research community has driven the emergence of various approaches for phenotyping the growth of plants by using either software or commercial equipment which one of their attributes is the determination of growth related measurements [46–49].

In this study, a semi-automatic methodology of estimating in a quantitative manner various morphological traits based on the image analysis tools is presented. Semi-automatic algorithms were created by applying intuitive mathematical descriptors with the aid of computing and image analysis software which quantify many features of fruit, leaf and endocarp. In particular, quantitative and qualitative characters of fruits, leaves and endocarps, such as size, shape, symmetry, contour roughness and presence of additional structures (nipple, petiole, etc.) were determined. We applied this methodology to describe olive organ characteristics, but, it can be easily used to describe the morphologies of other crop species, such as tomato, pear and grapevine.

## Methods

For various crops or model plant species, there are well established computational and computerized tools for plant phenotyping and morphological analysis; whereas for olive are limited or non-exstinded if non-extended. The aim of this study is to develop and unravel a semi-automated methodology, for rapidly providing reliable numerical values corresponding to morphological traits of olive fruits, leaves and endocarps. The proposed methodology consists of three main stages. The first stage is comprised of the collection of the olive fruit and leaf samples and their conversion to imaging data. The imaging data are images from olives fruits, leaves or endocarps and they include the necessary number of samples for the further analysis. The second stage is consisted of segmentation, in which the images of the objects (fruits, leaves, and endocarps) were separated from the background. At the third stage, the imaging data were fed to the advanced mathematical algorithms in order to perform the morphological analysis and the various traits be converted to numerical values. The interpretation of these values is very crucial to the motivation of this study.

### Description of samples

Fruit, leaf and endocarp samples of Greek olive cultivars such as Kalamon, Karidolia-Chalkidikis, Koroneiki, Lianomanako-Tyrou, Mastoidis, Megaron and Throumbolia were used. All the trees were grown under identical conditions, by the commercial nursery “Kostelenos” in Poros, Trizinia in Greece. Fruit samples at the breaker stage were collected from five trees of each cultivar. Samples of the fruits were collected from the mid-shoot portion of the current year’s growth from the most representative shoots, following a rotation around the tree at shoulder level (approximately 1.5 m from the ground level). They were collected on the external randomly chosen fruiting branches, avoiding large, small or irregular fruits and taking into account the fruit load on the trees; only trees with regular fruit load was considered. Moreover, mature, normally developed, leaves were collected from the most representative 1-year-old shoots on the south-facing side of the tree at shoulder level. Finally, the endocarps were extracted from the sample fruits and the pulp was removed by a coarse fabric. All kernels were soaked in $10\%$ bleach for 5 minutes and stored in a dry place, for later usage. At least 25 fruits, their endocarps and 25 leaves for each of the seven cultivars were analyzed.

### Imaging data

The imaging data of fruits and endocarps were created by using a $\textit{meopta}$ copy imaging stand. To avoid possible shadows, all the samples, were placed on the top of a lifted thin glass ($2\,\text {mm}$); whereas the camera was installed on the top of them, on a fixed solid arm. The light-blue paperboard was shown to be the most efficient background, for all the fruit and endocarps samples, as the better contrast was obtained. However, other colour paperboard can be used as background. Samples were lit with a set of ordinary bulbs with a correlated colour temperature (CCT) value of 5400 K; in which are supposed to provide the same or similar illumination as natural daylight. The leaves were scanned using a *HP DeskJet Ink Advantage 3636* scanner at a resolution at least 600 dpi (dots per inch). All the images were saved as jpeg or portable network graphics (png) files and a scaler was placed next to them.

**Fig. 1:**
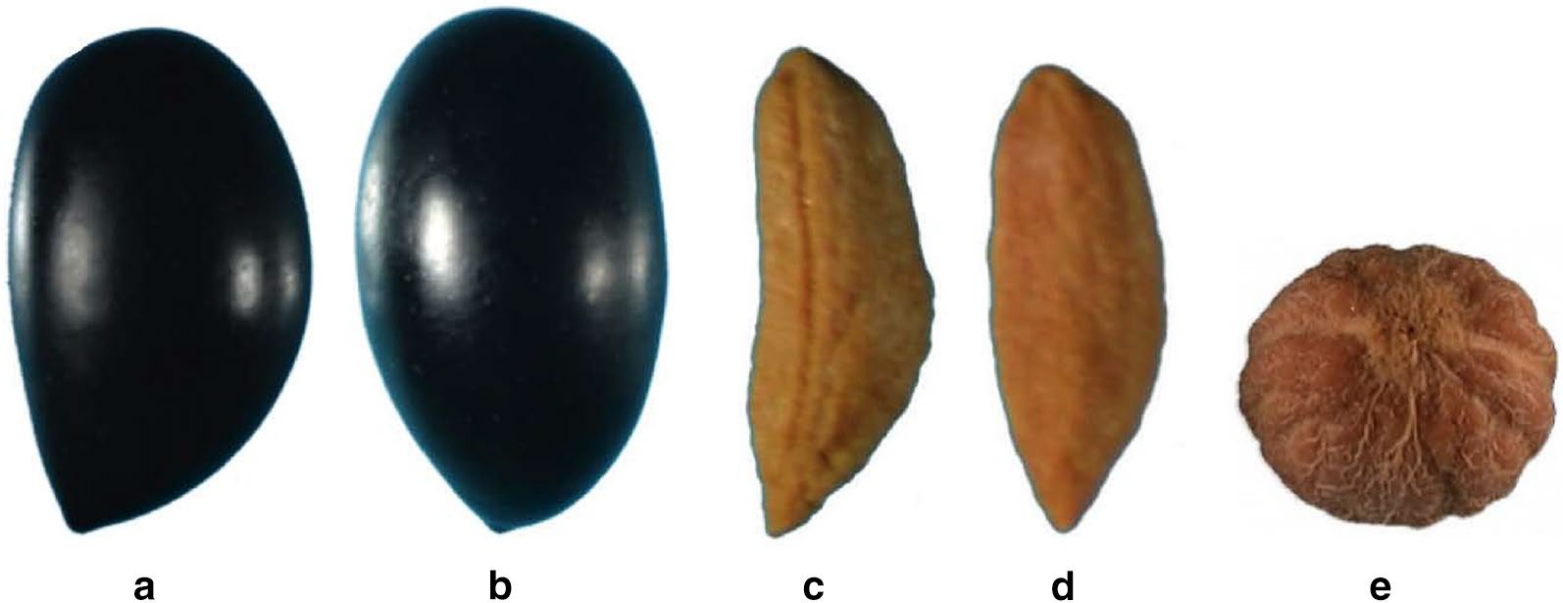
Different positions of olive fruits and endocarps. **a**–**c** Position-A of fruit and endocarp. They display their greatest asymmetry. For endocarps the carpel suture faces the observer. **b**–**d** Position-B of fruit and endocarps. It is reached by turning $90^{\circ }$ from A-position. **e** Position-C of endocarp; the petiole insertion is captured

Some of the morphological characters of fruits and endocarps refer to two imaging positions which are adopted by UPOV and International Olive Council (IOC). Position A refers to fruits or endocarps in which they display their greatest asymmetry; whereas, position B is reached by turning $90^{\circ }$ from position A (Fig. 1a–d). Also, the position A of the endocarp, is the one in which the carpel suture faces the observer. All fruits and endocarps were placed in the same orientation in which the base is upper than the apex. Furthermore to these usual positions, an additional vertical position has been considered for the morphological analysis of the endocarps. In this third position, the petiole insertion is captured and it is perfectly in the centre of each kernel’s image (Fig. 1e). The use of different positions in describing olive fruit or endocarp morphologies is indispensable, as the shape asymmetry and structure is taken into account and contribute to identify and classify olive cultivars.

### Image segmentation

**Fig. 2:**
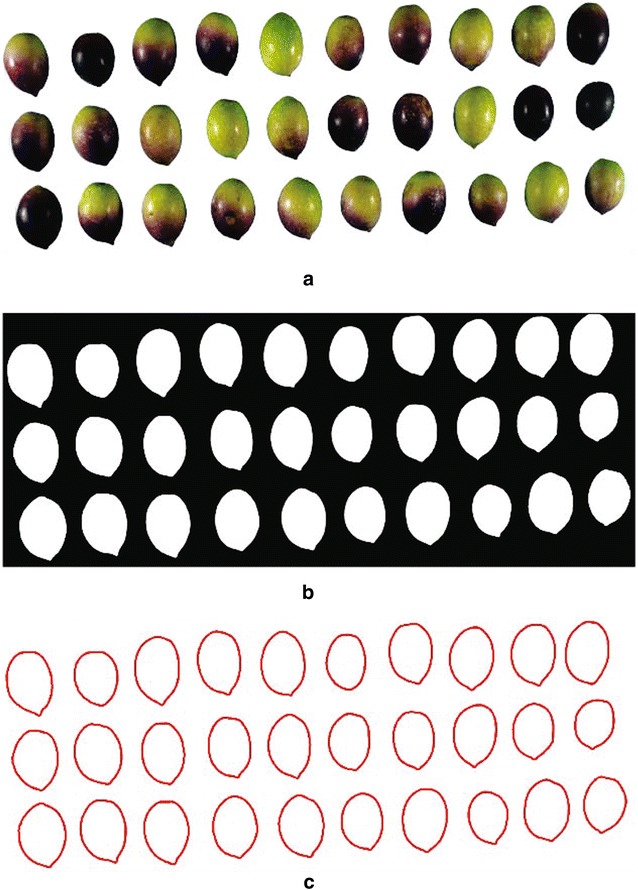
Pipeline of our methodology. **a** Raw imaging data from “Megaron” cultivar from “Kostelenos” olive nurseries in Poros–Trizinia, in Greece. **b** Segmented imaging data. A binary (black and white) image after the use of colour thresholding in *ImageJ*. **c** Shape boundaries. Representation of each shape by a discrete sequence with all its boundary points (red line)

Segmentation is the process during which the objects in an image are separated from the background [50]. Segmentation is one of the most important steps, from which a binary image (Fig. 2b) will be derived and then we will have the mathematical representation of each shape. The binarized image has only two values, either black or white. The black values corresponds to the background, whereas the white one to the describing object. The most commonly used methods for segmentation include thresholding, template matching and deformable models among others [51, 52]. In the present methodology, for the segmentation of the images, we are using *ImageJ* [24], which is one of the most commonly used open scientific image analysis software. We are using the colour thresholding technique, by modifying manually the values of “Hue”, “Saturation” and “Brightness” of each image. These values can be adjusted manually, in order to be found the best values for object segmentation. Nevertheless, the segmentation of the images can be performed with other different image analysis tools (e.g. Image-Pro Plus [53], Adobe Photoshop [54], etc.).

### Morphological analysis

The proposed methodology has been implemented in MATLAB (The Mathworks Inc., Natick, MA, USA) [55] and used the Image Processing Toolbox and created an automatic algorithm for object contour extraction from the binary images (Fig. 2) and identify various geometrical characteristics which are assigned to different morphological traits. The object of interest is olive fruit, leaf or endocarp.

The final outcome of the algorithm is the representation of each shape by a discrete sequence with all its boundary points. This sequence with the boundary points represents each shape and it can be considered mathematically, as a closed polygonal line, which vertices are the boundary points and its (undirected) edges were defined by subsequent vertices, additionally, connecting the last with the first vertex (Fig. 2c). This mathematical representation of the experimental data enables to analyze quantitatively and qualitatively the morphology of the olives, leaves and endocarps of each cultivar.

As we mentioned, earlier the segmentation step results in a binary image in which each shape (in white) is separated by its background (in black). Due to image resolution issues the resulting shape is appearing to have embossed and irregular edges. We apply a function on the boundary that makes the boundary to be smoother and has a more physical appearance. This technique is applicable to each shape (from olive leaves, fruits and endocarps) before its further morphological analysis.

The algorithm we propose for describing fruit morphology may now be stated in pseudocode as follows:

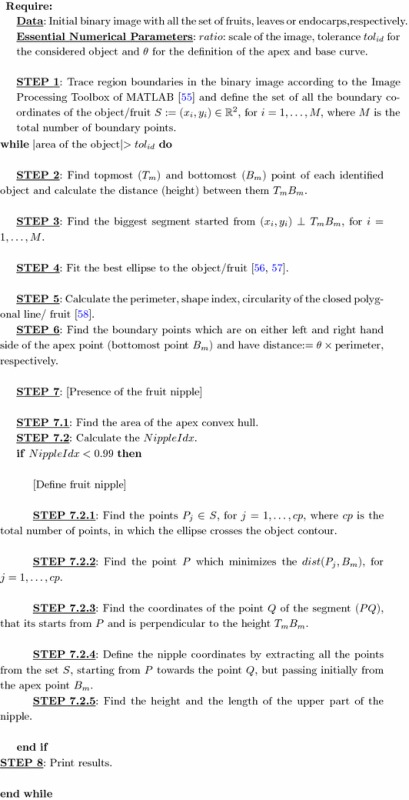



The algorithmic part for the morphological analysis of the leaves and the endocarps has a similar workflow.

**Fig. 3:**
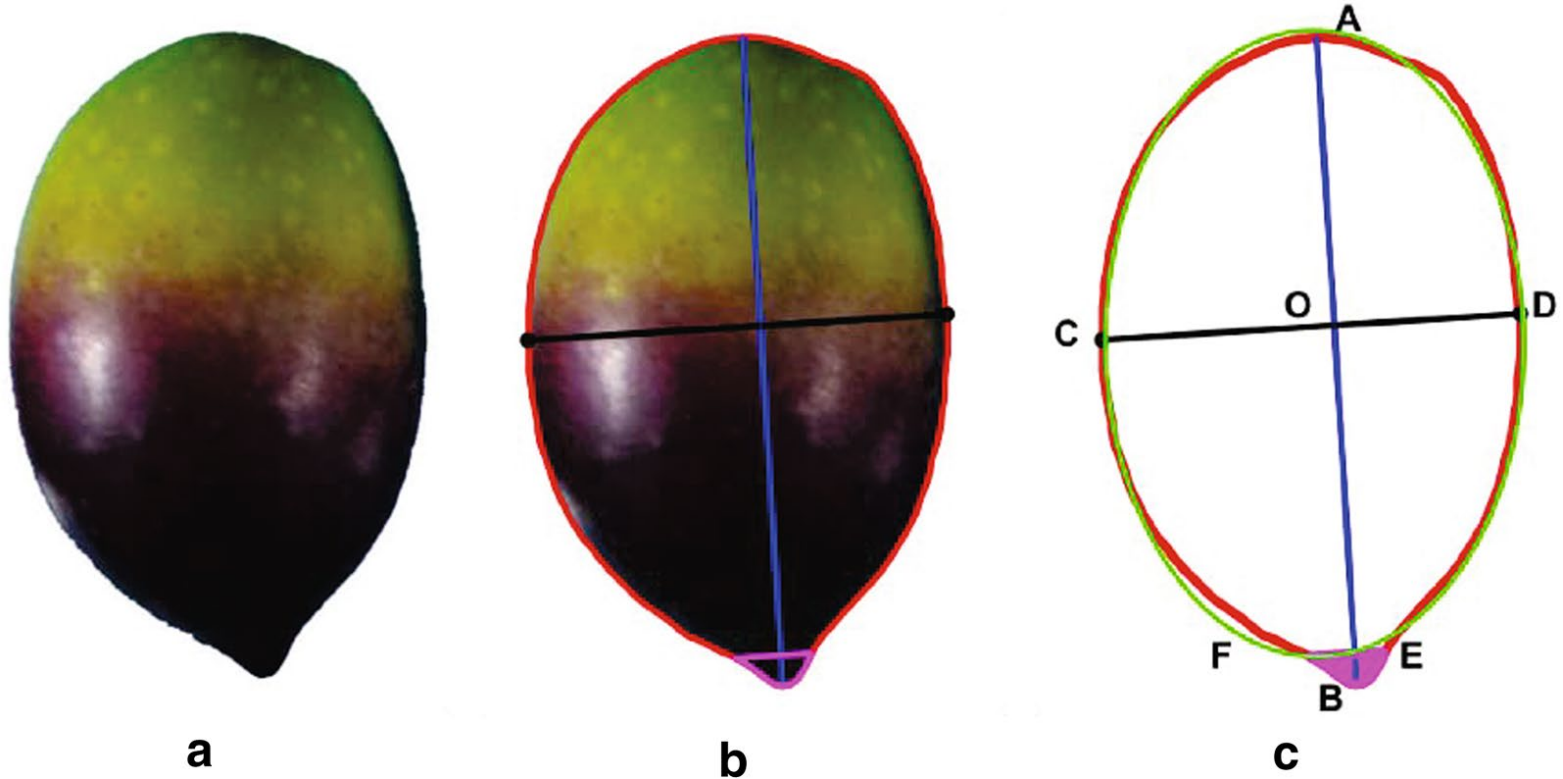
Morphological characters of the fruit. **a** Raw image data. **b** Raw image data with measurements. **c** Morphological measurements. Contour (red line) fruit boundary, height (*A*–*B*): (blue line), maximum transverse diameter (*C*–*D*) (black line), position of the maximum transverse diameter: segment (*O*–*B*), best fit ellipse: green curve, fruit nipple: pink curve (arc (EBF))

For the better morphological description of the fruit shape, we considered the characters which describe as much as possible its geometry. Thus, the following shape features have been taken into account for both described positions (Fig. 3):

- Area, perimeter, height, maximum transverse diameter and **position of maximum transverse diameter**.
- Vertical and transversal symmetry.
- Major and minor axis of a fitted ellipse, **shape index**.
- **Nipple presence** and the geometry of the nipple (height, area and the length of the upper part of the nipple).
- **Shape of apex and base/nipple**.

The morphological descriptors which are in bold are related to cultivar identification [21–23].

**Fig. 4:**
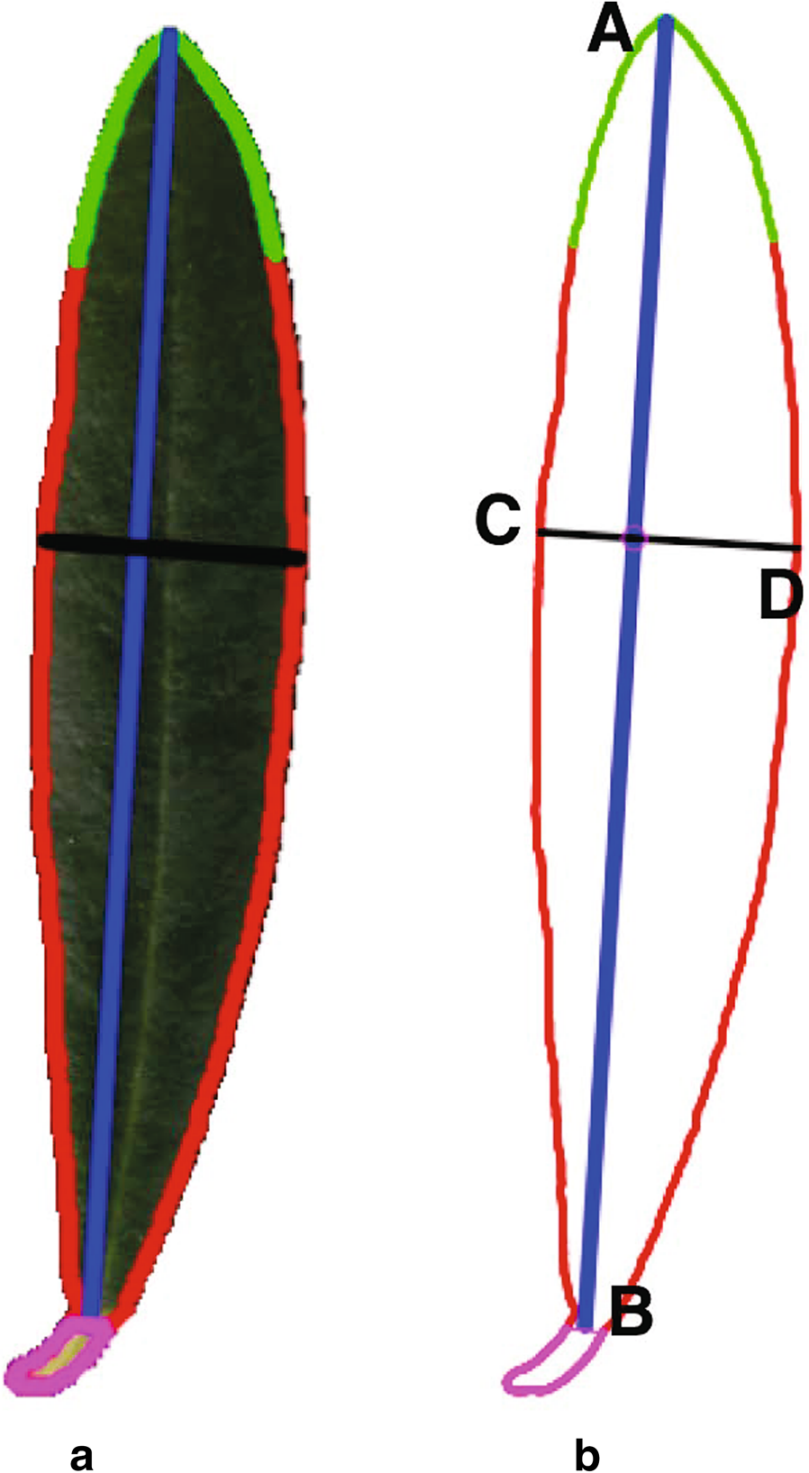
Morphological characters of a leaf. **a** Raw image data. **b** Morphological measurements. Contour (red line): leaf boundary, Tip curve: green line, blade height: segment (A–B)-(blue line), width: segment (C–D)-(black line), petiole: pink curve

Similarly, for the description of leaf morphology, we considered the following parameters, that are suggested by the UPOV (Fig. 4):

- Area, perimeter.
- Height and width of the leaf blade.
- **Shape index**.

Additionally and complementary to the above measurements, we included the following characters for describing the leaf morphology:

- **Position of the width**.
- Vertical and transversal symmetry.
- Major and minor axis of a fitted ellipse.
- **Shape of tip**.
- Height and area of the petiole.

The endocarp is the internal, woody part of the olive fruit that encloses the seed. Usually the word “stone” refers to the endocarp and seed together. The morphological analysis of the endocarps is of great importance, as their morphological traits are less-influenced by the environmental condition and the training systems [2, 6, 11]. The endocarp is the most suitable organ compared to fruit and leaf for cultivar identification. Most likely, the endocarp is the less affected organ by environmental conditions due to its wooden nature, the protection from the olive pulp coverage and its brief exposure to the environmental conditions [11, 12, 16].

**Fig. 5:**
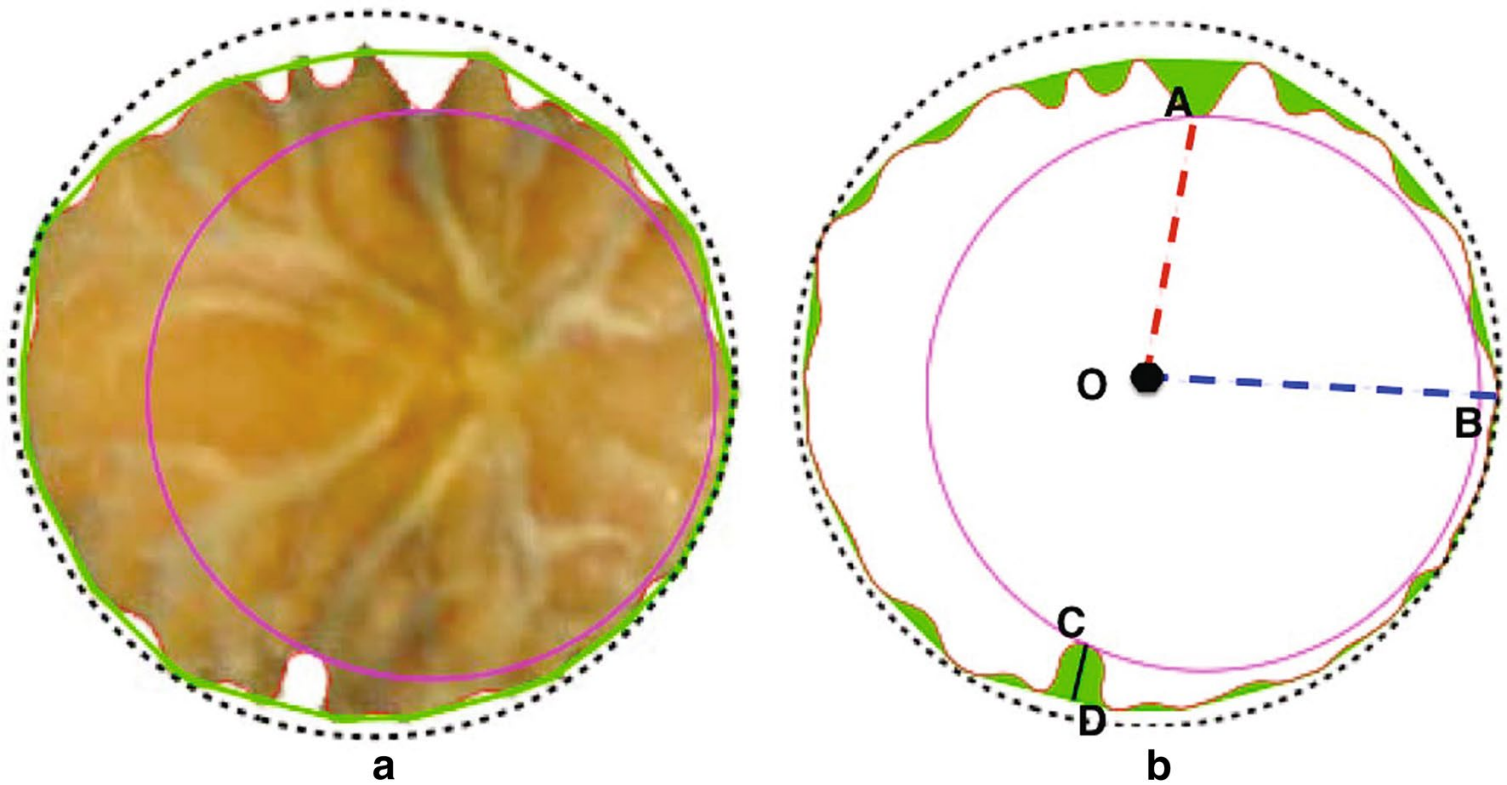
Morphological characters of the endocarp in vertical position. **a** Raw image data. **b** Morphological measurements. Contour (red line) endocarp boundary, convex hull polygon (green line), green area area between the endocarp and its convex hull, maximum depth of the grooves (C–D) (black line vertical to the polygonal edge), shortest distance from the centre to the endocarp boundary segment (O–A)-(red dashed line), longest distance from the centre to the endocarp boundary segment (O–B)-(blue dashed line), inscribed circle the largest circle inside the endocarp boundary (pink dashed circle), minimum bounding circle (black dashed circle)

As we mentioned earlier, in this study, we have considered a supplementary vertical position facing the base of the endocarp. From this vertical position, the study of critical endocarps features becomes more robust and efficient, in a semi-automatic way. Here, as with olive fruits and leaves, apart the size and shape features, we considered additionally from the vertical position the following traits (Fig. 5):

- **Surface roughness**.
- Maximum and minimum distance from the centre to the contour.
- The diameter of the best fit circle.
- **Average and maximum depth of the grooves**.

Summarizing, for the morphological analysis of fruit we used 24 parameters that describe fruit morphological characteristics; whereas for the leaf we used 16. Finally, for the stone’s morphology description in the position A and B we used 22 morphological characters and for the vertical position we considered 22, additionally. All the morphological characters are purely mathematical defined and the most representatives are appeared and explained briefly in Table 1.

All the morphological characters that we considered in this methodology are presented in the Additional file 1. In the Additional file 2 we present all the necessary description for all the morphological traits above and their definitions.

**Table 1:** List of the most representatives morphological characters that are described within the current methodology

Parameter	Description
Area $(\text {cm}^2)$	Area inside the polygonal line-contour
Perimeter (cm)	Total length of the polygonal line-contour
Hght (cm)	Height: length between the topmost and bottomost point of each contour
MaxTrDiam (cm)	Longest segment perpendicular to the height
MinCntTr (cm)	Minimum distance between the height and the contour in MaxTrDiam
VerSym	Describes the ratio of the position of the MaxTrDiam by the height
TrSym	Describes the ratio of the MinCntTr by the MaxTrDiam
ShIdx	Shape Index: Height/MaxTrDiam
ApCur	Mean curvature of the apex
BasCur	Mean curvature of the base
Circ	Circularity: dimensionless shape descriptor based on the contour perimeter
NippleIdx	Presence or not of a nipple in a fruit (1:YES, 0:NO)
BladeHgt (cm)	Length of the leaf without the petiole
TipCur	Mean curvature of the leaf tip curve
StArConv	Area convexity; describes the surface roughness of the endocarp
MinCentCnt (cm)	Minimum distance between the centre of mass and the contour
MaxCentCnt (cm)	Largest distance between the centre of mass and the contour
DiamInscrCir (cm)	Largest diameter of the inscribed circle
DiamMinBdCir (cm)	Largest diameter of the minimum bounding circle

## Results

### Morphological analysis and cultivar identification

The numerical data defining quantitatively a morphological trait of olive fruit, leaf or endocarp of seven Greek olive cultivars were analyzed and post-processed using the statistics toolbox of MATLAB (The Mathworks Inc., Natick, MA, USA) [55]. The standard descriptive statistical methods were applied on the numerical findings and the data re expressed as mean ± standard errors (SEs) (Table 2). Then one-way analysis of variances (one-way ANOVA) was applied on the data of the morphological parameters, to analyze and determine possible statistically significant differences in the data sets (Table 3). Furthermore, we used a multicomparison approach (Tukey’s honest significant difference criterion-95$\%$ confidence interval) to identify the differences between each pair of cultivars.

**Fig. 6:**
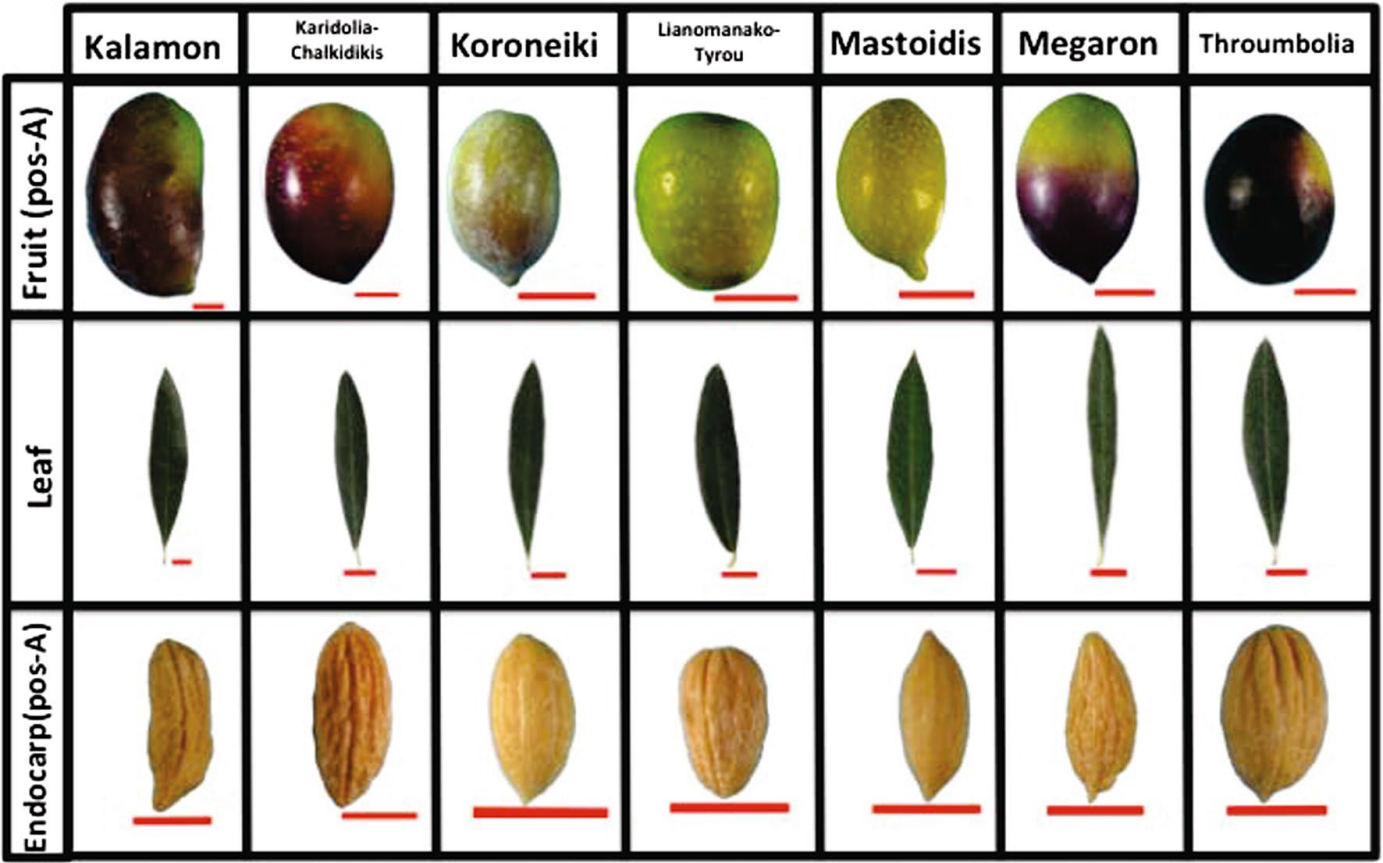
Greek olive-samples. Representative olive fruit, leaf and endocarp of the Greek olive cultivars characterized in this proof-of-concept morphological analysis, using the proposed methodology. Red line represents 1*cm*

The comparison of the fruit area average values in position A indicated that Karidolia-Chalkidikis had larger fruits; whereas Koroneiki, Lianomanako-Tyrou and Mastoidis were clustered into the small fruit group (Fig. 6). Moreover, we detected that Kalamon, Koroneiki and Mastoidis had elongated fruits, while Karidolia-Chalkidikis and Megaron were more elliptic according to the shape index. In addition, olive fruits of Lianomanako-Tyrou and Throumbolia had a more spherical shape. The current methodology appeared to successfully detect the presence of the nipple in olive fruits. Thus, the nipple index was positive only for the Koroneiki, Mastoidis and Megaron cultivars. The description of the transversal and vertical symmetry was also precisely detected with the current methodology.

Kalamon appeared to be the only cultivar with the largest leaf, whereas Mastoidis and Throumbolia had smaller leaf according to the leaf morphological analysis (Fig. 6). The leaf of Megaron cultivar was the only one with a lanceolate shape while the blade width in all cultivars appeared to be of medium size. Moreover, the tip shape appeared to be almost the same for the Koroneiki, Lianomanako-Tyrou and Throumbolia.

**Table 2:** Morphological parameters of seven Greek olive cultivars

	Morphological parameter	Kalamon Cultivar1	Karidolia-Chalkidikis Cultivar2	Koroneiki Cultivar3	Lianomanako-Tyrou Cultivar4	Mastoidis Cultivar5	Megaron Cultivar6	Throubolia Cultivar7
Fruit	Area-A	5.51 ± 0.108	8.87 ± 0.113	1.55 ± 0.101	2.78 ± 0.101	2.76 ± 0.101	4.56 ± 0.101	4.72 ± 0.108
	Perim-A	8.76 ± 0.095	10.81 ± 0.099	4.63 ± 0.088	6.01 ± 0.088	6.34 ± 0.088	7.81 ± 0.088	7.79 ± 0.095
	Hght-A	3.31 ± 0.04	3.92 ± 0.04	1.77 ± 0.03	2.06 ± 0.03	2.49 ± 0.03	2.91 ± 0.03	2.72 ± 0.04
	MaxTrDiam-A	2.06 ± 0.024	2.91 ± 0.025	1.18 ± 0.023	1.67 ± ± 0.023	1.49 ± 0.023	2.06 ± 0.023	2.21 ± 0.024
	minCntTr-A	0.92 ± 0.017	1.34 ± 0.017	0.53 ± 0.015	0.74 ± 0.015	0.63 ± 0.015	0.93 ± 0.015	1.04 ± 0.017
	TrSym-A	0.408 ± 0.007	0.4447 ± 0.007	0.451 ± 0.006	0.449 ± 0.006	0.399 ± 0.006	0.436 ± 0.006	0.474 ± 0.007
	ShIdx-A	1.597 ± 0.015	1.346 ± 0.015	1.501 ± 0.014	1.237 ± 0.014	1.670 ± 0.014	1.412 ± 0.014	1.232 ± 0.015
	Circ-A	0.896 ± 0.003	0.952 ± 0.003	0.911 ± 0.002	0.965 ± 0.002	0.859 ± 0.002	0.936 ± 0.002	0.974 ± ± 0.003
	ApCur-A	0.013 ± 0.001	0.010 ± 0.001	0.029 ± 0.0009	0.009 ± 0.0009	0.042 ± 0.0009	0.023 ± 0.0009	0.007 ± 0.001
	BasCur-A	0.008 ± 0.0004	0.007 ± 0.0004	0.012 ± 0.0004	0.009 ± 0.0004	0.011 ± 0.0004	0.010 ± 0.0004	0.007 ± 0.0004
	NippleIdx-A	No	No	Yes	No	Yes	Yes	No
	ShIdx-B	1.629 ± 0.016	1.343 ± 0.016	1.524 ± 0.014	1.253 ± 0.014	1.670 ± 0.014	1.403 ± 0.014	1.221 ± 0.016
	VerSym-B	0.442 ± 0.007	0.462 ± 0.007	0.433 ± 0.014	0.006 ± 0.006	0.461 ± 0.006	0.481 ± 0.006	0.475 ± 0.007
Leaf	Area	11.646 ± 0.216	4.938 ± 0.203	4.363 ± 0.203	4.289 ± 0.199	3.593 ± 0.189	4.700 ± 0.196	3.924 ± 0.207
	Perimeter	20.806 ± 0.229	13.413 ± 0.215	12.556 ± 0.215	12.004 ± 0.211	10.958 ± 0.200	14.633 ± 0.207	11.490 ± 0.220
	ShIdx	4.816 ± 0.098	5.287 ± 0.093	4.827 ± 0.093	4.802 ± 0.091	4.594 ± 0.086	6.338 ± 0.089	4.371 ± 0.094
	MaxTrDiam	1.894 ± 0.028	1.129 ± 0.027	1.148 ± 0.027	1.115 ± 0.026	1.053 ± 0.025	1.026 ± 0.026	1.145 ± 0.027
	VerSym	0.460 ± 0.009	0.497 ± 0.009	0.509 ± 0.009	0.499 ± 0.009	0.490 ± 0.008	0.560 ± 0.008	0.511 ± 0.009
	TipCur	0.004 ± 0.0002	0.005 ± 0.0002	0.006 ± 0.0002	0.006 ± 0.0002	0.007 ± 0.0002	0.005 ± 0.0002	0.006 ± 0.0002
	Circ	0.334 ± 0.006	0.345 ± 0.005	0.347 ± 0.005	0.371 ± 0.005	0.374 ± 0.005	0.274 ± 0.005	0.373 ± 0.006
Endocarp	Area-A	1.412 ± 0.028	1.772 ± 0.028	0.575 ± 0.027	0.828 ± 0.027	0.772 ± 0.027	1.006 ± 0.027	1.100 ± 0.027
	Perim-A	5.019 ± 0.061	5.400 ± 0.061	2.929 ± 0.058	3.463 ± 0.058	3.688 ± 0.058	4.145 ± 0.058	4.000 ± 0.058
	TrSym-A	0.273 ± 0.011	0.404 ± 0.011	0.418 ± 0.010	0.453 ± 0.010	0.345 ± 0.010	0.382 ± 0.010	0.421 ± 0.010
	ShIdx-A	2.516 ± 0.033	2.236 ± 0.033	1.859 ± 0.032	1.755 ± 0.032	2.466 ± 0.032	2.220 ± 0.032	1.817 ± 0.032
	ApCur-A	0.030 ± 0.001	0.024 ± 0.001	0.038 ± 0.001	0.024 ± 0.001	0.0358 ± 0.001	0.033 ± 0.001	0.028 ± 0.001
	BasCur-A	0.026 ± 0.001	0.018 ± 0.001	0.028 ± 0.001	0.014 ± 0.001	0.037 ± 0.001	0.039 ± 0.001	0.021 ± 0.001
	Circ-A	0.704 ± 0.006	0.764 ± 0.006	0.842 ± 0.006	0.867 ± 0.006	0.713 ± 0.006	0.735 ± 0.001	0.855 ± 0.006
	VerSym-B	0.398 ± 0.011	0.486 ± 0.011	0.462 ± 0.010	0.436 ± 0.010	0.522 ± 0.010	0.530 ± 0.010	0.521 ± 0.010
	ShIdx-B	2.406 ± 0.030	2.009 ± 0.030	1.891 ± 0.029	1.776 ± 0.029	2.548 ± 0.029	2.244 ± 0.029	1.706 ± 0.029
	StArConv-C	0.984 ± 0.004	0.967 ± 0.007	0.996 ± 0.0008	0.984 ± 0.003	0.993 ± 0.002	0.975 ± 0.002	0.989 ± 0.002
	DiamIncrCir-C	0.885 ± 0.016	1.001 ± 0.016	0.618 ± 0.016	0.745 ± 0.016	0.651 ± 0.016	0.762 ± 0.016	1.002 ± 0.016

Next to each parameter we refer the position in which either the fruit or the endocarp is placed for morphological analysis. The results are presented in the form mean ± SE

*A* position A, *B* position B and *C* position C

**Table 3:** Statistical analysis of morphological data

	Morphological parameter	ANOVA (p-values)	Multiple comparison Tukey–Kramer
Fruit	Area-A	< 0.0001	1$\ne$2$\ne$3$\ne$4$\ne$5
	Perim-A	< 0.0001	1$\ne$2$\ne$3$\ne$4$\ne$7
	Hght-A	< 0.0001	1$\ne$2$\ne$3$\ne$4$\ne$5$\ne$6$\ne$7
	MaxTrDiam-A	< 0.0001	1$\ne$2$\ne$3$\ne$4$\ne$5$\ne$7
	minCntTr-A	< 0.0001	1$\ne$2$\ne$3$\ne$4$\ne$5$\ne$7
	TrSym-A	< 0.0001	5$\ne$6$\ne$7
	ShIdx-A	< 0.0001	1$\ne$2$\ne$3$\ne$4$\ne$5$\ne$6
	Circ-A	< 0.0001	1$\ne$2$\ne$3$\ne$4$\ne$5$\ne$6
	ApCur-A	< 0.0001	1$\ne$3$\ne$5$\ne$6$\ne$7
	BasCur-A	< 0.0001	2$\ne$3$\ne$4
	NippleIdx-A	< 0.0001	–
	ShIdx-B	< 0.0001	1$\ne$2$\ne$3$\ne$7
	VerSym-B	< 0.0001	3$\ne$5
Leaf	Area	< 0.0001	1$\ne$2$\ne$7
	Perimeter	< 0.0001	1$\ne$2$\ne$4$\ne$5$\ne$6
	ShIdx	< 0.0001	1$\ne$2$\ne$6$\ne$7
	MaxTrDiam	< 0.0001	1$\ne$3$\ne$6
	VerSym	< 0.0001	1$\ne$3$\ne$6
	TipCur	< 0.0001	1$\ne$2$\ne$4$\ne$5
	Circ	< 0.0001	3$\ne$5$\ne$6
Endocarp	Area-A	< 0.0001	1$\ne$2$\ne$3$\ne$4$\ne$6$\ne$7
	Perim-A	< 0.0001	1$\ne$2$\ne$3$\ne$5$\ne$6
	TrSym-A	< 0.0001	1$\ne$2$\ne$4$\ne$5
	ShIdx-A	< 0.0001	2$\ne$3$\ne$5
	ApCur-A	< 0.0001	2$\ne$3$\ne$6$\ne$7
	BasCur-A	< 0.0001	3$\ne$4$\ne$5$\ne$7
	Circ-A	< 0.0001	1$\ne$2$\ne$3$\ne$4$\ne$6
	VerSym-B	< 0.0001	1$\ne$3$\ne$5
	ShIdx-B	< 0.0001	1$\ne$2$\ne$4$\ne$5$\ne$6
	StArConv-C	< 0.0001	1$\ne$2$\ne$3
	DiamIncrCir-C	< 0.0001	1$\ne$2$\ne$5$\ne$6$\ne$7

The one-way ANOVA showed that all the morphological parameters of the seven cultivars were statistically different (p-values $<\,0.0001$). The morphological parameters discriminated either all or some of the seven olive cultivars as revealed by the Tukey–Kramer multiple comparisons

Koroneiki and Mastoidis had small endocarps, while Karidolia-Chalkidikis and Kalamon had larger according to the morphological analysis of the endocarps (Fig. 6). Moreover, the endocarps of Lianomanako-Tyrou had an ovoid shape whereas Koroneiki and Throumbolia were more elliptic based on the shape index of the endocarp in position A. The shape of the endocarps of Kalamon, Karidolia-Chalkidikis, Mastoidis and Megaron appeared to be elongated. This methodology also successfully detected the surface roughness from the vertical position. The surface of the endocarp of Karidolia-Chalkidikis was more scabrous, whereas those of Koroneiki, Mastoidis and Throumbolia were smoother after analysis of the numerical values.

The one-way ANOVA showed that all the morphological parameters of the seven cultivars were statistically different (p-values $<0.0001$). Moreover, all the parameters discriminated either all or some of the seven olive cultivars as revealed by the Tukey–Kramer multiple comparisons. This allowed the initiation of an additional investigation regarding the utility of this methodology for olive cultivar identification and discrimination using only the computed morphological parameters. For example, the multiple comparisons of the fruit shape index in position A, which was related to cultivar identification appeared to discriminate successfully six out of the seven olive cultivars.

### Morphological analysis of other crop species

**Fig. 7:**
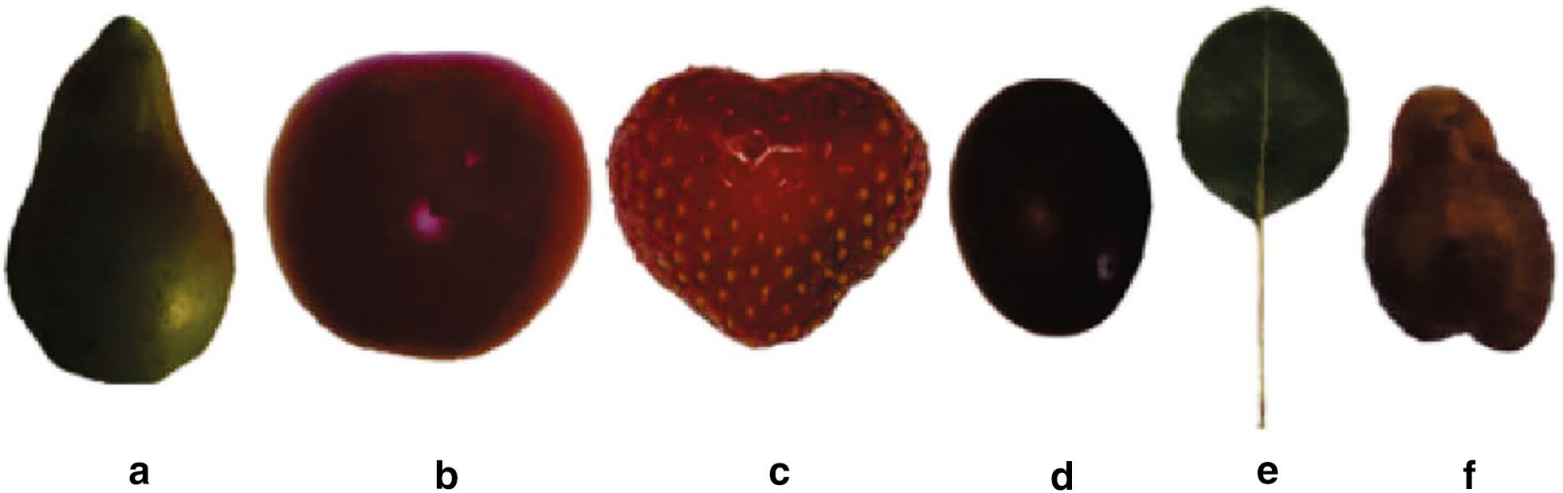
Morphological analysis of other crop species. The methodology can easily describe morphologies of other crop species. Here we applied on **a** pear, **b** tomato, **c** strawberry, **d** grape-berry, **e** pear leaf and **f** grapevine seed

The methodology was implemented in other crop species such as tomato, pear, strawberry and grapevine in order to assess its applicability for the morphological characterization of various plant organs (Fig. 7). Boundaries of plant organs were successfully determined and various traits (such as height, width, area, apex shape etc.) were calculated in tomato, grapevine, pear and strawberry fruit, as well as pear leaf and grapevine seed (Table 4). These results indicate that this methodology can be used in other plant species and organs.

**Table 4:** Morphological analysis of crop species

	ShIdx	Area (cm$^2$)	MaxTrDiam (cm)	VerSym
Pear	1.633	8.829	2.832	0.350
Tomato	1.033	9.466	3.478	0.489
Strawberry	1.093	7.487	3.308	0.321
Grape berry	1.145	4.322	2.208	0.574
Pear leaf	1.276	18.256	4.397	0.525
Grapevine seed	1.504	1.307	0.918	0.561

Selected numerical values from morphological traits of a pear, tomato, strawberry, grapevine, pear leaf and grapevine seed. Images of all these appear in Fig. 7

## Discussion

The morphological characterization is still considered the first choice for the description and classification of the olive germplasm, based on either traditional time-consuming or on software-based methodologies. For the olive breeders or plant biologists, the description of the fruit, leaf or endocarp morphology is of significant importance for phenomics studies.

*ImageJ* is widely used for numerical data generation of morphological parameters. Important measurements for the characterization of an organ shape, such as the position of the maximum transverse diameter or the apex shape required either manual determination or advanced programming skills. Thus, the measurements with *ImageJ* required manual, time-consuming procedures which sometimes were leading to insufficient results.

The *Tomato Analyser* required digital images of sliced in half fruits being placed in black background [30]. These prerequisites made this software unsuitable for olive morphology studies due to high labor intensive needs. The Leaf Analyser program was limited to perform morphological analysis only on plant leaves, despite its robustness and effectiveness while the Elliptic Fourier method, which was based on purely geometrical and mathematical notions, failed to calculate and quantify accurately useful traits associated with shape morphology [33, 41, 42]. Finally, other commercial equipment or platforms which offer phenomics analysis were developed mostly for studying the growth of plants. Therefore, their aim was differed compared to our objective [46–49].

The comparison of our approach with the available software and methodologies indicated that the interesting feature was the semi-automated morphological analysis of fruits, leaves and endocarps without pre-processing manual tasks such as cutting the fruits or prerequisites regarding the background of the image after a manual binarization (black & white) of the initial image. The proposed methodology was based on robust mathematical descriptors which could provide more accurate, rapid and consistent results regarding the shape description.

The morphological analysis of the olive cultivars is based on the mathematical representation of the boundary of each shape (fruit, leaf or endocarp) by a polygonal line and considering it as a mathematical shape. The examination is purely based on the geometrical and mathematical properties of these shapes-polygonal curves. Therefore, using fundamental shape descriptors we found a neat and robust methodology to detect and describe as much as possible information, of either the fruit, leaf or endocarp morphologies, in a semi-automatic and numerically precise manner. The intention was not to conduct any multivariate approach to determine possible relationships among the morphological parameters or perform cluster analysis. The statistical analysis of the data showed the applicability of the methodology towards olive cultivar identification and discrimination. The morphological combined with genetic analyses are efficient for olive germplasm management. Thus, this methodology could assist in the direction of the construction of international olive germplasm databases by determining their morphological parameters.

### Current limitations and future development

The development of this methodology is the first step in the direction of describing olive cultivars using image analysis tools in a semi-automated manner. The caveats and the current limitations of this methodology is the bias imposed by the participation of the user. For example, for the separation of fruits, leaves and endocarps from the image background, we are using the manual colour thresholding technique, which is commonly used in many applications in plants. However, this procedure can be less biased by the user, by using interactive segmentation methods or recent developed tools for such purposes [49, 59]. Moreover, the proposed methodology does not allow the user to interact within the workflow and calculate a parameter such as the determination of the apex point of a fruit which was attached to the pedicel. Finally, as this methodology is only available in the MATLAB environment, future programming work is required in order to be developed in open programming libraries. From computational and developmental point of view, our approach to detect the fruit nipple is based on a threshold value that has been determined by trial-and error, and even small threshold deviations may affect its detection. Although the sensitivity of this approach, it could be a stepping stone in the direction of developing a future automated framework for fruit nipple detection.

The numerical values are calculated by only considering the boundary and mathematical representation of the contour of either the fruit, leaf or endocarp morphology. In the future, additional qualitatively measurements of attributes regarding the colour of the olive fruit, leaf or endocarp will be considered. An additional study is under way in which the effect of different environmental conditions in three consecutive years in the morphology of different genotypes will be assessed in order to further validate the methodology for cultivar identification. Besides the proposed methodology is developed for the two-dimensional morphological analysis of olive cultivars, a future direction is its extension to the three-dimensional setting. Although, extending this framework to three-dimensional settings is not at all trivial, it can directly become a less biased methodology as the 3D morphology of the fruit, leaf or endocarp will be considered.

## Conclusion

In this study, a further step towards the development of an integrated, more comprehensive automated methodology was presented for describing fruits, leaves and endocarps morphologies. Moreover, it provided more accurate and objective numerical measurements regarding the olive morphology attributes, which were rather difficult to be measured manually or by other available computational tools or software. Meaningful morphological traits were presented to describe fruit, leaf or endocarp morphologies. The parameters had been defined strictly mathematically leading to a more robust and efficient methodology. Meaningful morphological traits were presented to describe fruit, leaf or endocarp morphologies. The developed parameters have been defined strictly mathematically, and this gives to our methodology the opportunity to be more robust and efficient. Nevertheless, many issues still remain open and need further investigation.

Finally, the proposed methodology might be considered a useful, rapid and reliable image-based tool to identify and discriminate olive cultivars. This initial attempt was performed using profile pictures of fruits, leaves and endocarps of Greek olive cultivars. A future use of this tool, which will take into account different microclimates and orchard growth management practices, will assess its performance for cultivar identification. This user friendly methodology is a useful step towards the morphological analysis of crop species; especially olives, and will be available to scientists and researchers from different areas of plant biology.

## Additional files

**Additional file 1.** Morphological parameters extracted from the proposed methodology.

**Additional file 2.** Definitions of the morphological parameters.

## Abbreviations

Hght: height
MaxTrDiam: maximum transverse diameter
MinCtTr: minimum distance between the height and the contour in MaxTrDiam
VerSym: vertical symmetry
TrSym: transversal symmetry
ShIdx: shape index
ApCur: mean curvature of the apex
BasCur: mean curvature of the base
Circ: cicularity
NippleIdx: presence or not of a fruit nipple
BladeHgt: height of leaf blade
TipCur: mean curvature of the tip
StArConv: area convexity
MinCentCnt: minimum distance between the centre of mass and the contour
MaxCentCnt: maximum distance between the centre of mass and the contour
DiamInscrCir: largest diameter of the inscribed circle
DiamMinBdCir: largest diameter of the minimum bounding circle
