# Supplementary material for: Description of olive morphological parameters by using open access software
Source: Plant Methods. 2017 Dec 11;13:111. doi: 10.1186/s13007-017-0261-8 (PMC5725956; doi:10.1186/s13007-017-0261-8)
Supplement: Supplementary file 1 — Additional file 1. Morphological parameters extracted from the proposed methodology. [file 13007_2017_261_MOESM1_ESM.pdf]

Morphological Traits extracted from the new methodology

FRUIT (A & B position)

1) Area
2) Perimeter
3) Height
4) Maximum transverse diameter
5) Minimum distance between the transversal diameter and the contour
6) Vertical Symmetry
7) Transversal Symmetry
8) Shape Index
9) Major axis of the ellipse
10) Minor axis of the ellipse
11) Curvature of the convex hull of the apex
12) Curvature of the convex hull of the base
13) Circularity of the fruit (ci_fruit).
14) Circularity of the ellipse (ci_ell).
15) Circularity ratio (ci_fruit/ci_ell)
16) Ratio of area of the shape by the area of its convex hull
17) Presence or not of a fruit nipple (1:YES;0:NO)
18) Nipple height
19) Area of the nipple
20) Distance of the upper part of the nipple
21) Mean curvature of the nipple
22) Height ratio (nipple height/height of fruit)
23) Area ratio (nipple area/area of fruit)
24) Angle of the nipple

LEAF

1) Area
2) Perimeter
3) Height
4) Maximum transverse diameter
5) Minimum distance between the transversal diameter and the contour
6) Vertical symmetry
7) Transversal symmetry
8) Shape index

9) Major axis of the ellipse
10) Minor axis of the ellipse
11) Petiole height
12) Area of the petiole
13) Distance of the upper part of the petiole
14) Curvature of the apex
15) Curvature of the base or petiole
16) Circularity of the leaf

Endocarp (A& B Position)

1) Area
2) Perimeter
3) Height
4) Maximum transverse diameter (maxTrDia)
5) Minimum distance between the transversal diameter and the contour
6) Vertical Symmetry
7) Transversal Symmetry
8) Shape Index
9) Major axis of the ellipse
10) Minor axis of the ellipse
11) Area of the apex curve
12) Area of the base curve
13) Length of the segment that links the two end points of the apex (dist_ap)
14) Length of the segment that links the two end points of the base (dist_bas)
15) Curvature of the apex
16) Curvature of the base
17) Circularity of the endocarp (ci_endocarp)
18) Circularity of the ellipse (ci_ell)
19) Circularity ratio (ci_obj/ci_ell)
20) Length ratio (apex) (dist_ap/maxTrDia)
21) Length ratio (base) (dist_bas/maxTrDia)
22) Mean curvature of the convex hull of the apex
23) Ratio of areas base/area of its convex hull
24) Mean curvature od the convex hull of the apex
25) Ratio of area apex/area of its convex hull

ENDOCARP VERTICAL (C position)

1) Area
2) Perimeter
3) Area of convex hull
4) Area between the convex hull and the contour
5) Ratio of area of the shape by the area of its convex hull
6) Mean vertical distance from each contour point to the closest convex hull segment
7) Maximum vertical distance from each contour point to the closest convex hull segment
8) Mean distance from the centre of the object to its boundary
9) Minimum distance from the centre of the object to its boundary
10) Maximum distance from the centre of the object to its boundary
11) Circularity of the contour (ci_obj)
12) Circularity of the convex hull(ci_ch)
13) Circularity ratio (ci_obj/ci_ch)
14) Diameter of the best fit circle
15) Diameter of the inscribed circle
16) Area of the inscribed cirle
17) Diameter of the minimum bounding circle
18) Area of the minimum bounding circle
19) Mean distance between the convex hull points
20) Max distance between the convex hull points
21) Min distance between the convex hull points
