# Supplementary material for: Description of olive morphological parameters by using open access software
Source: Plant Methods. 2017 Dec 11;13:111. doi: 10.1186/s13007-017-0261-8 (PMC5725956; doi:10.1186/s13007-017-0261-8)
Supplement: Supplementary file 2 — Additional file 2. Definitions of the morphological parameters. [file 13007_2017_261_MOESM2_ESM.pdf]

Supplementary File 2

1 Morphological analysis

Characters of the fruits

Definition 1 (**Contour**). *Contour S is a closed polygonal line, consisting of M points, that defines the shape boundary (red line in Figure 3 in the manuscript)*

$$S := \{(x_i, y_i)|x_i, y_i \in \mathbb{R}\}, \tag{1}$$

with $i = 1, \dots, M$, and $(x_1, y_1) = (x_M, y_M)$.

Definition 2. *Perimeter of the contour is defined as*

$$Perimeter := \sum_{i=1}^{M-1} \left((x_{i+1} - x_i)^2 - (y_{i+1} - y_i)^2\right)^{\frac{1}{2}}. \tag{2}$$

Definition 3 (**Height**). *The height is defined as the Euclidean distance between the two toppmost and bottomost point of each contour (blue line (AB) in Figure 3 in the manuscript)*

$$(AB) = \left((x_T - x_B)^2 + (y_T - y_B)^2\right)^{\frac{1}{2}}, \tag{3}$$

where $T_m := (x_T, y_T)$ and $B_m := (x_B, y_B)$ is the toppmost and bottomost point, respectively.

This height definition appears to be more effective and robust than the one that is commonly used in other computational tools or software; in this the height is defined as the distance of the two far apart points of the contour [1, 2, 3].

Definition 4 (**Maximum transverse diameter**). *The biggest segment which is perpendicular to the height (black line (CD) in Figure 3 in the manuscript).*

Definition 5 (**Position of maximum transverse diameter**). *The position of the maximum transverse diameter is given by the distance of the apex to the crossing point of the height and the maximum transverse diameter (the segment (OB) in Figure 3 in the manuscript).*

Definition 6 (**Vertical symmetry**). *The vertical symmetry of the fruit is defined as the ratio of the position of the maximum transverse diameter by the height*

$$vertical\ symmetry = \frac{position\ of\ the\ maximum\ transverse\ diameter}{fruit\ height} = \frac{(OB)}{(AB)}. \tag{4}$$

Definition 7 (**Transversal symmetry**)**.** *The transversal symmetry of the fruit is defined as the ratio of the minimum distance of the crossing point of the height to the contour (MinCntTr) by the length of the maximum transverse diameter (Figure 3 in the manuscript)*

$$transversal\ symmetry = \frac{MinCntTr}{maximum\ transverse\ diameter} = \frac{CO}{CD}. \quad (5)$$

Definition 8 (**Fitted ellipse**)**.** *The fitted ellipse is the best ellipse that can describe the shape (green curve in Figure 3 in the manuscript) [4, 5]. The parameters that describe an ellipse are its major and minor axes.*

Definition 9 (**Shape index**)**.** *The shape index is a unitless parameter that it is defined as the ratio of the height by the maximum transverse diameter,*

$$shape\ index = \frac{height}{maximum\ transverse\ diameter} = \frac{AB}{CD}. \quad (6)$$

According to the UPOV, the shape of the fruit can be grouped (Figure 3 in the manuscript) based on the shape index, as follow:

$$shape\ index = \begin{cases} spherical & if\ < 1.25cm, \\ elliptic & if\ 1.25 - 1.45cm, \\ elongated & if\ > 1.45cm. \end{cases} \quad (7)$$

Definition 10 (**Apex curve**)**.** *The apex curve of the fruit is defined by its apex point (bottomost point in the methodology) and by considering a curve with length that equals with a certain distance proportionally of the fruit perimeter (i.e. $\theta \times perimeter$) on either left and right hand side, respectively. In our case, this certain distance was determined by trial-and-error and the best value chosen is $\theta = \frac{1}{8}$.*

Definition 11 (**Presence or absence of fruit nipple**)**.** *The presence or absence of the fruit nipple can be defined by the comparison of the area of the apex curve and its convex hull polygon. Thus, we consider the following ratio*

$$NippleIdx := \frac{Area\ of\ the\ apex\ curve}{Area\ of\ the\ apex\ convex\ hull\ polygon} \quad (8)$$

and we conclude

$$Detection\ of\ a\ fruit\ nipple\ := \begin{cases} Presence\text{-}Yes & if\ NippleIdx < 0.99, \\ Absence\text{-}No & if\ NippleIdx > 0.99. \end{cases} \quad (9)$$

The threshold value 0.99 that is used in the above definition for the nipple presence on a fruit, has been determined by trial-and-error.

Definition 12 (**Fruit nipple**)**.** *The fruit nipple is defined by the closest point (point F in Figure 3 in the manuscript) to the apex (point B in Figure 3 in the manuscript) crosses the ellipse and the contour. Then, the vertical line from this point to the height crosses the contour on the opposite side in a point (point E in Figure 3 in the manuscript), which finally defines the boundary (EBF) of the nipple (pink curve in Figure 3 in the manuscript).*

Definition 13 (**Nipple height**). *The nipple height is defined as the distance from the apex to the line that bounds the nipple (segment (EF) in Figure 3 in the manuscript).*

Definition 14 (**Length of the upper part of the nipple**). *The length of the upper part of the nipple is defined as the distance of the segment that bounds the nipple (segment (EF) in Figure 3 in the manuscript).*

Definition 15 (**Circularity**). *It is a dimensionless shape factor based on the perimeter of the contour boundary [6] and represents how the shape is similar to a circle.*

Characters of the leaves

The definitions of the leaves related traits are identical with those that we have described earlier for the fruit characters. Thus, we refer to them. Regarding the definition of the petiole, and therefore the definition of the leaf blade, we are using the same technique/procedure as the one for the fruit nipple. Moreover, similarly with the fruit apex curve, we defined the leaf tip as the polygonal curve, which is defined by its topmost point, and by considering a curve that equals with $\frac{1}{12}$ of the leaf perimeter on either left and right hand side, respectively.

Figure 4 in the manuscript, shows a representation of the above measurements in raw imaging data.

Characters of the endocarps

In this study, we have considered a supplementary vertical position facing the base of the endocarp. From this vertical position, the study of critical endocarps features becomes more robust and efficient, in a semi-automatic way. Here, as with olive fruits and leaves, apart the size and shape features, we considered additionally from the vertical position the following traits:

- **Surface roughness**.
- Maximum and minimum distance from the centre to the contour.
- The diameter of the best fit circle.
- **Average and maximum depth of the grooves**.

To evaluate the surface roughness, we invented a neat methodology that can be applied for its better description.

Definition 16 (**Surface roughness**). *The surface roughness is determined by its area convexity and can be defined by the following ratio (Figure 5 in the manuscript)*

$$StArConv := \frac{Area_{endocarp}}{Area_{convex\ hull}}. \quad (10)$$

Definition 17 (**Average and maximum depth of the grooves**). *The average and maximum depth of the grooves can be measured by the mean and the longest vertical distance of all the contour points between two consecutive points of the convex hull of the endocarp, to the corresponding straight line, respectively (Figure 5 in the manuscript).*

Finally, taking into account the endocarp size, we found more efficient to describe the apex curve by the apex point (bottomost point) by considering a curve with length that equals the $\frac{1}{25}$ of the endocarp perimeter on either their left and right hand side. Similarly we define the base curve.

Bibliography

References

[1] M. D. Abràmoff, P. J. Magalhães, and S. J. Ram, "Image processing with ImageJ," *Biophotonics international*, vol. 11, no. 7, pp. 36–42, 2004.

[2] M. T. Brewer, L. Lang, K. Fujimura, N. Dujmovic, S. Gray, and E. van der Knaap, "Development of a controlled vocabulary and software application to analyze fruit shape variation in tomato and other plant species," *Plant physiology*, vol. 141, no. 1, pp. 15–25, 2006.

[3] T. Tanabata, T. Shibaya, K. Hori, K. Ebana, and M. Yano, "SmartGrain: high-throughput phenotyping software for measuring seed shape through image analysis," *Plant physiology*, vol. 160, no. 4, pp. 1871–1880, 2012.

[4] R. Halır and J. Flusser, "Numerically stable direct least squares fitting of ellipses," in *Proc. 6th International Conference in Central Europe on Computer Graphics and Visualization. WSCG*, vol. 98, pp. 125–132, 1998.

[5] A. Fitzgibbon, M. Pilu, and R. B. Fisher, "Direct least square fitting of ellipses," *IEEE Transactions on pattern analysis and machine intelligence*, vol. 21, no. 5, pp. 476–480, 1999.

[6] M. Yang, K. Kpalma, and J. Ronsin, "A survey of shape feature extraction techniques," *Pattern recognition*, pp. 43–90, 2008.
